# Change in Body Mass Index is Associated with Change in Cognition in Older Adults

**DOI:** 10.1093/geroni/igaa057.3239

**Published:** 2020-12-16

**Authors:** Joy Douglas, Kristi Crowe-White, Amy Ellis, Chuong Bui, Saroja Voruganti, Kristine Yaffe

**Affiliations:** 1 The University of Alabama, Tuscaloosa, Alabama, United States; 2 University of Alabama, Tuscaloosa, Alabama, United States; 3 University of North Carolina at Chapel Hill, Kannapolis, North Carolina, United States; 4 UCSF Weill Institute for Neurosciences, San Francisco, California, United States

## Abstract

Background: Alzheimer’s disease and related dementias affect one in ten Americans age 65y and older. Considering the rapid growth of the aging population, identifying modifiable risk factors for cognitive decline is a public health priority. Although weight change later in life is common, its impact on cognition is unclear. The objective of this study was to examine the relationship between change in body mass index (BMI) and cognition among older adults. Methods: The Health, Aging, and Body Composition Study was a prospective study of community-dwelling adults ages 70-79y at baseline (n=3,075; 49% males, 42% African-American). Using baseline and year 10 visit data, we evaluated change in BMI and change in cognition measured by the Modified Mini-Mental Status Exam (3MS) using a linear mixed model. Change in 3MS scores were regressed on changes in time-varying BMI after controlling for blood pressure, glucose, cholesterol, race, education, biological sex, and APOE genotype. Results: At baseline, average BMI was 27.4 (n=3075) and average 3MS was 90.1 (n=3061). At year 10, average BMI was 27.1 (n=1600) and average 3MS was 88.6 (n=1598). Higher BMI was associated with less cognitive decline (ceteris paribus). This finding suggests that weight gain is associated with cognitive maintenance. The effect of an increase in BMI was largest for those underweight at baseline. Conclusion: Among underweight older adults, an increase in BMI may be desirable for maintaining cognition. Although more research is needed, these findings suggest the need for interventions to prevent unintentional weight loss among older adults.

